# Reaching Out to Adolescents at High Risk of HIV Infection in Brazil: Demand Creation Strategies for PrEP and Other HIV Combination Prevention Methods

**DOI:** 10.1007/s10508-022-02371-y

**Published:** 2022-07-25

**Authors:** Laio Magno, Fabiane Soares, Eliana Miura Zucchi, Marcos Eustórgio, Alexandre Grangeiro, Dulce Ferraz, Dirceu Greco, Maria Mercedes Escuder, Ines Dourado, The PrEP1519 Study Group

**Affiliations:** 1 Departamento de Ciências da Vida, Universidade do Estado da Bahia, Rua Silveira Martins, 2555, Cabula41.150-000, Salvador, Bahia Brazil; 2 Instituto de Saúde Coletiva, Universidade Federal da Bahia, Salvador, Bahia Brazil; 3 Programa de Pós-Graduação em Saúde Coletiva, Mestrado Profissional em Psicologia e Políticas Públicas, Universidade Católica de Santos, São Paulo, Santos Brazil; 4 Faculdade de Medicina, Universidade de São Paulo, São Paulo, São Paulo Brazil; 5 Escola FIOCRUZ de Governo, Fundação Oswaldo Cruz, Brasília, Distrito Federal Brazil; 6 Faculdade de Medicina, Universidade Federal de Minas Gerais, Belo Horizonte, Brazil; 7 São Paulo State Health Department, Institute of Health, São Paulo, São Paulo Brazil

**Keywords:** HIV pre-exposure prophylaxis, Adolescent, Demand creation, Men who have sex with men, Transgender women, Brazil

## Abstract

Using baseline data from the PrEP1519 cohort, in this article we aimed to analyze: (i) the effectiveness of demand creation strategies (DCS) to enroll adolescent men who have sex with men (AMSM) and adolescent transgender women (ATGW) into an HIV combination prevention study in Brazil; (ii) the predictors of DCS for adolescents’ enrollment; and (iii) the factors associated with DCS by comparing online and face-to-face strategies for enrollment. The DCS included peer recruitment (i.e., online and face-to-face) and referrals from health services and non-governmental organizations (NGOs). AMSM and ATGW who agreed to participate in the study could opt to enroll in either PrEP (PrEP arm) or to use other prevention methods (non-PrEP arm). Bivariate and multivariate analyses were conducted and logistic regression odds ratios were estimated. The DCS reached 4529 AMSM and ATGW, the majority of which were derived online (73.8%). Of this total, 935 (20.6%) enrolled to participate (76.6% in PrEP arm and 23.4% in non-PrEP arm). The effectiveness of enrolling adolescents into both arms was greater via direct referrals (235/382 and 84/382, respectively) and face-to-face peer recruitment (139/670 and 35/670, respectively) than online (328/3342). We found that a combination under DCS was required for successful enrollment in PrEP, with online strategies majorly tending to enroll adolescents of a higher socioeconomic status. Our findings reinforce the need for DCS that actively reaches out to all adolescents at the greatest risk for HIV infection, irrespective of their socioeconomic status.

## Introduction

Youth and adolescents from low- and middle-income countries (LMIC) face barriers to HIV prevention (Patton et al., 2016) and are exposed to multiple factors that increase their risk for HIV infection. Such factors may operate at the individual level (e.g., low level of education, use of psychoactive substances, difficulty in talking about sexuality at one’s home and/or at school) (Felisbino-Mendes et al., 2018; Jarrett et al., 2018; Magno et al., 2022;), the programmatic level (e.g., limited availability of HIV prevention services, exigence of parental consent for consultations) (DeMaria et al., 2009; Magno et al., 2022), and the structural level (e.g., conservative environment, absence of legislative protection against sexual coercion, violence, and discrimination for adolescents) (Dubov et al., 2018; Magno et al., 2022, 2019a, b; Melesse et al., 2020).

HIV infections are increasing among adolescents in different regions of the world, including LMIC (UNAIDS, 2020). In Latin America, the epidemic is concentrated among key populations, particularly men who have sex with men (MSM) and transgender women (TGW) (UNAIDS, 2020). In Brazil, surveys using respondent-driven sampling (RDS) showed a high-HIV prevalence among adults MSM (Kerr et al., 2013, 2018) and TGW (Bastos et al., 2018; Grinsztejn et al., 2017).

In the last decade, the AIDS incidence rate among males aged 15 to 19 years in Brazil has increased from 3.7 cases per 100,000 inhabitants in 2009 to 6.1 in 2019—an increase of 64.9% (Brasil, 2020a). Despite the scarcity of data regarding HIV prevalence among adolescent men who have sex with men (AMSM) in Brazil, the available data seems to draw a disproportional rate for this population when compared to adolescents from the general population (Coelho et al., 2021; Saffier et al., 2017). For example, the official surveillance data on the HIV seroprevalence among male adolescents in Brazil demonstrates that over time, AMSM have a higher HIV prevalence in comparison to heterosexual boys; furthermore, there is an increasing trend among adolescents and young MSM aged 17 to 22 years old: 0.56% in 2002 (Szwarcwald et al., 2005), 1.23% in 2007 (Szwarcwald et al., 2011) and 1.32% in 2016 (Sperhacke et al., 2018). Regarding adolescent transgender women (ATGW), to the best of our knowledge, data on the burden of the HIV epidemic in this group is still lacking.

HIV combination prevention strategies have been proposed to reduce infection rates, including pre-exposure prophylaxis (PrEP), with recent data regarding its effectiveness and feasibility from different countries such as South Africa (Celum et al., 2019), USA (Hosek et al., 2017a, b; Hosek et al., 2017a, b), and Brazil (Dourado et al., 2020). Nevertheless, expanding PrEP access and uptake among youth and adolescents remains a challenge (Jaspan et al., 2011; Ongwandee et al., 2018; Tolley et al., 2014). Services need to be youth-friendly, inclusive, and employ strategies to increase demand by delivering positive messaging about the benefits of PrEP as a component of HIV combination prevention (Celum et al., 2019; Dourado et al., 2020).

In the last decade, several strategies to boost demand targeting youth and adolescents (Maloney et al., 2020) have been used in demonstration studies, clinical trials, and health services aiming to increase the coverage and effectiveness of HIV prevention control measures (Ongwandee et al., 2018). Such strategies include peer recruitment (Lightfoot et al., 2018; Ongwandee et al., 2018), peer-facilitated community-based interventions (Rose-Clarke et al., 2019), interventions on online platforms (Du Bois et al., 2012), and mobile apps (Ippoliti & L’Engle, 2017; Sullivan & Hightow-Weidman, 2021), among others.

Despite these efforts, studies analyzing the effectiveness of strategies used for demand creation are still scarce, especially concerning adolescents at greater risk for HIV infection (Bradley et al., 2020). To contribute to filling this gap, we aimed to analyze: i) the effectiveness of demand creation strategies (DCS) to enroll AMSM and ATGW into an HIV combination prevention study in Brazil; ii) the predictors of DCS for adolescents’ enrollment, and iii) the factors associated with DCS by comparing online and face-to-face strategies for enrollment.

## Method

### Study Design and Population

DCS were employed as part of a demonstration project titled PrEP1519, which aimed at analyzing the effectiveness of PrEP and other HIV combination prevention methods among AMSM and ATGW aged between 15 and 19 years, ongoing in PrEP clinics across three capital cities in Brazil: Belo Horizonte (located at a youth reference center), Salvador (located at a Diversity Center that advocates for Lesbian, Gay, Bisexual, Transgender, Queer, Intersexual and other LGBTQI + rights), and São Paulo (located in an HIV testing and counseling center). All peer educators (young MSM or TGW) and health professionals working on the demonstration project were trained. The DCS were implemented from February 2019 to February 2021.

### DCS and Data Collection

A summary of the DCS can be found in Table 1. These strategies were the same across all three sites, while respecting the cultural differences of each region.

**Table 1:** PrEP1519 DCS for AMSM and ATGW

Strategy	Platform	Approach/Contextualization
*Online DCS*		
Amanda Selfie—chatbot	Facebook	Amanda Selfie–Brazil's first transgender chatbot–can be accessed on Facebook, where she talks to young people about the project and PrEP, using artificial intelligence. When an individual is eligible for the study, the chatbot administers the recruitment questionnaire and schedules an appointment. She interacts with adolescents, provides sex education, and enables them to link up to the PrEP clinics or other HIV testing and care services. Site link: https://www.facebook.com/amandaselfie.bot
Peer-educator recruitment on dating/“hook-up” apps	Grindr, Tinder, Badoo, and Scruff	In dating apps, using a PrEP1519 project profile, peer educators actively recruit young people from the target population who are interested in PrEP. The peer educator presents the project and explains the importance of HIV prevention. When an individual is interested in participating in the project, the peer educators administer the recruitment questionnaire and invite them to visit the clinic. In Grindr and Scruff, the project managed to capture more MSM, while on Badoo there were more transgender girls. Tinder is quite diverse. Dating apps are indicated for young adults, so we recruited more youth aged 18 and 19 years old
Peer-educator recruitment on social media	Instagram	The project has an Instagram profile on which it actively disseminates content on LGBTQI + sexual health and HIV prevention, especially PrEP. It is an important communication channel with young people, through which it is possible to ask questions and make an appointment at the project clinic. The peer educators also actively recruit young people from the target population who interact with our Instagram profile. When they identify an eligible individual, who is open to dialogue, the peer educators present the project, administer the recruitment questionnaire, explain the importance of HIV prevention, and invite them to visit the clinic. On Instagram, it was possible to reach a greater number of minors aged 15 to 17
Peer-educator recruitment on social media	WhatsApp	The project’s WhatsApp contact details are on all its publicity materials and its social media. Young people who are interested in participating in the study can contact the team and schedule their appointment at the clinic via this channel

Strategy	Platform	Approach/contextualization
*Face-to-face DCS*		
Peer-educator recruitment	Social venues, schools, non-governmental organizations (NGO), and parties	The LGBTQI + youth team used specific cultural codes to provide information about PrEP and sexual health and prevention materials. Peer education led by adolescents and youth works systematically and periodically with groups of adolescents in previously mapped social venues (e.g., bars, parks, beaches, streets). At the time of the actions, educational and prevention materials were delivered. Face-to-face strategies, in general, have higher costs due to expenses related to transportation, food, and, in some cases, added infrastructure
NGO	Partner NGO	Partner NGOs referred young people at risk for HIV infection who are eligible for the project
Direct referrals	Health services; friends and/or sexual partners	Referrals were made by health professionals from public services when they identified young people eligible to use PrEP; and by study participants or friends who knew about the project from the demand creation actions

Approaching and communicating with a target population is a key element for the success of any DCS (Bradley et al., 2020). Aiming to map out where AMSM and ATGW hung out in each city, the implementation of the PrEP clinics was preceded by a formative research, which employed direct observation of social venues, and in-depth interviews and focus groups with key informants (Zucchi et al., 2021). Besides mapping the venues, interviews and focus groups were employed to explore adolescents’ prior knowledge and acceptability of PrEP, their sexual behavior and HIV and sexually transmitted infections (STI) prevention behavior, and their opinion about the DCS planned by the study to reach out ATGW and AMSM.

The DCS developed by the study included face-to-face and online approaches. Both included discussions regarding sexual orientation and gender identity, sexual behavior, and HIV prevention, as well as the distribution of HIV self-test (HIVST), condoms, lubricants, and douching supplies (in the case of online approaches, the prevention supplies were sent to participants by mail or picked up at PrEP services, according to participants’ preference). From mid-March of 2020 onward, face-to-face activities were adapted to online activities due to the COVID-19 pandemic (Dourado et al., 2020).

All adolescents who accessed the DCS received pertinent information about the study, and those who agreed to participate signed an assent or a consent form as needed. Those presenting a high risk of or vulnerability to HIV infection (i.e., unprotected anal sex in the last 6 months, previous episode of STI or use of HIV post-exposure prophylaxis (PEP) in the last 12 months, frequent use of alcohol or drugs before or during sexual intercourse, transactional or commercial sex, or experiences of discrimination and violence) were invited to enroll in the PrEP clinics. After evaluation from a provider, participants were able to choose, with assistance from the provider, which of the study arms they would like to be enrolled in: (i) the PrEP arm, which included daily use of oral PrEP along with the TDF/FTC combination, or (ii) the non-PrEP arm, in which participants who were PrEP-eligible but chose not to use PrEP had access to other HIV combination prevention methods (i.e., counseling, condoms, lubricant, douche, PEP, and HIVST). In both cases, quarterly follow-ups included medical consultations, HIV and STI testing, counseling, and access to prevention supplies. Participants received a reimbursement to cover transportation expenses.

All AMSM and ATGW, aged between 15 and 19 years, identified through DCS (i.e., online, face-to-face peer recruitment, direct referrals, and NGO) were invited to answer a recruitment questionnaire. When the participants arrived at the PrEP clinics for enrollment, they were invited to answer a more detailed socio-behavioral questionnaire (e.g., demographics, sexual behavior, drug use, STI, discrimination, and violence). For this analysis, data from both of these questionnaires were used.

Data from the two questionnaires were recorded by the peer educator or health provider in an online database platform. The recruitment questionnaire had a unique automatic code that was linked by the health provider to the socio-behavioral questionnaire when the participant arrived at the PrEP clinic, thereby preventing duplication. Adolescents who were approached more than once were categorized using the information from the first contact such individuals could be identified when they used the same social media profile or when they provided information at PrEP clinics.

### Study Variables

**Fig. 1:**
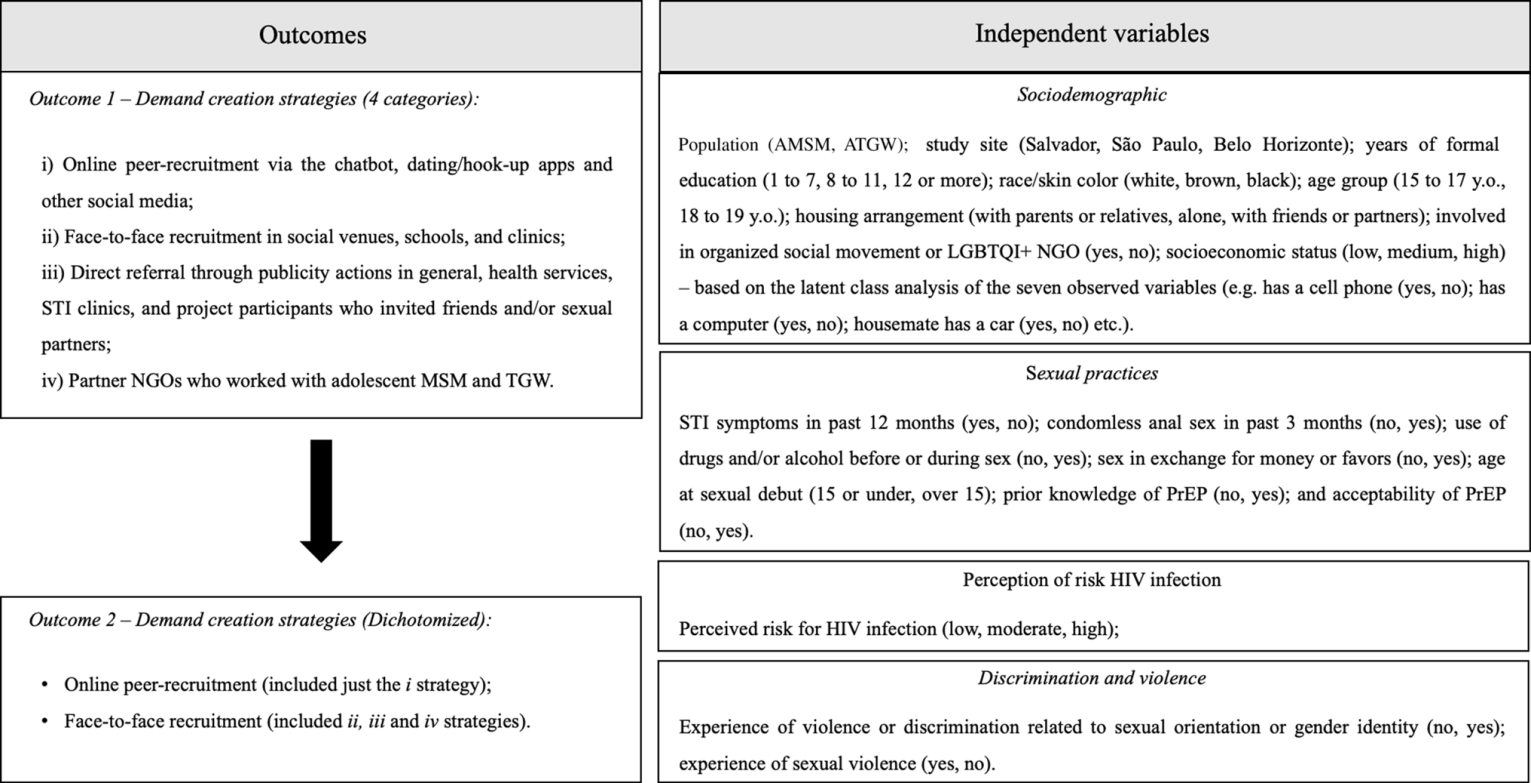
Study variables in PrEP1519 study, Brazil (2019–21)

The outcomes were as follows: (i) DCS across four categories (i.e., online, face-to-face peer recruitment, direct referrals, and NGO); and (ii) DCS dichotomized into “online” (i.e., only online recruitment) and “face-to-face” (i.e., peer recruitment face-to-face, direct referrals, and NGO) (Fig. 1).

Socioeconomic status (low, middle, and high) was based on the latent class analysis (LCA) of the following variables: having sought employment in the last month (yes, no); having a landline at home (yes, no); owning a cell phone (yes, no); owning a computer (yes, no); having access to the internet (yes, no); housemate having a car (yes, no); employing a house cleaner at home (yes, no). To identify the best LCA model, the Akaike information criterion and the Schwarz–Bayesian information criterion were used, assuming the lowest values to provide the best fit. Moreover, model selection was based on the parsimony and separation of the classes. Other predictor variables are presented in Fig. 1.

### Data Analysis

Firstly, we conducted a descriptive analysis of the DCS based on the recruitment questionnaire across four categories (i.e., online, face-to-face peer recruitment, direct referrals, and NGO), stratified into time periods before and during the COVID-19 pandemic across the three cities (mid-March 2020). In the bivariate analysis, we initially analyzed predictors of DCS (four categories) for adolescents’ enrollment, based on the socio-behavioral questionnaire, and stratified by the study arms. Fisher exact tests were used to test the associations. In the multivariate analysis, DCS was recategorized as (i) “online” (i.e., only online recruitment) and (ii) “face-to-face” (i.e., peer recruitment face-to-face, direct referrals, and NGO). Multivariate analysis was conducted using logistic regression models, estimating the adjusted odds ratios (aOR) for factors associated with DCS and comparing online and face-to-face strategies for enrollment. To select the variables to be included in the logistic regression model, the P value was set at ≤ 0.20 in the bivariate analysis. After this, the variables were selected based on the literature review and statistical significance (*p* < 0.05) using backward elimination, and with satisfactory residual analysis, maintained within the final model. The fit of the model was assessed using the Hosmer–Lemeshow goodness-of-fit test. These analyses were conducted using the program R, version 4.0.3.

### Ethical Consideration and Approval

This study was conducted in accordance with the guidelines from the Brazilian Research Ethics Commission Resolution 466/2012. The protocol was approved by the Research Ethics Committees of the World Health Organization (Protocol ID: Fiotec-PrEP Adolescent study) and the three Brazilian universities at each site (USP, UFBA, and UFMG). Written informed consent (WIC) was sought and obtained from the adolescents aged 18 and 19 years old. For those under 18, each city followed a different protocol, as per local court decisions: in Belo Horizonte, the WIC had to be signed by the parents or guardian, followed by the assent form (AF) signed by the adolescents; in Salvador, there were two possibilities: (i) WIC signed by a parent or guardian and AF by the adolescent; or (ii) just AF signed by the adolescent, in which case the team’s psychologist and social worker judged that their family ties had been broken or that they were at risk of physical, psychological, or moral violence due to their sexual orientation; and in São Paulo a judicial decision allowed a waiver of parental consent, which is why just the AF signed by the adolescents was enough to be able to join the study. All participants could withdraw participation at any stage of the process or skip any questions they perceived as too sensitive, personal, or distressing.

## Results

**Fig. 2:**
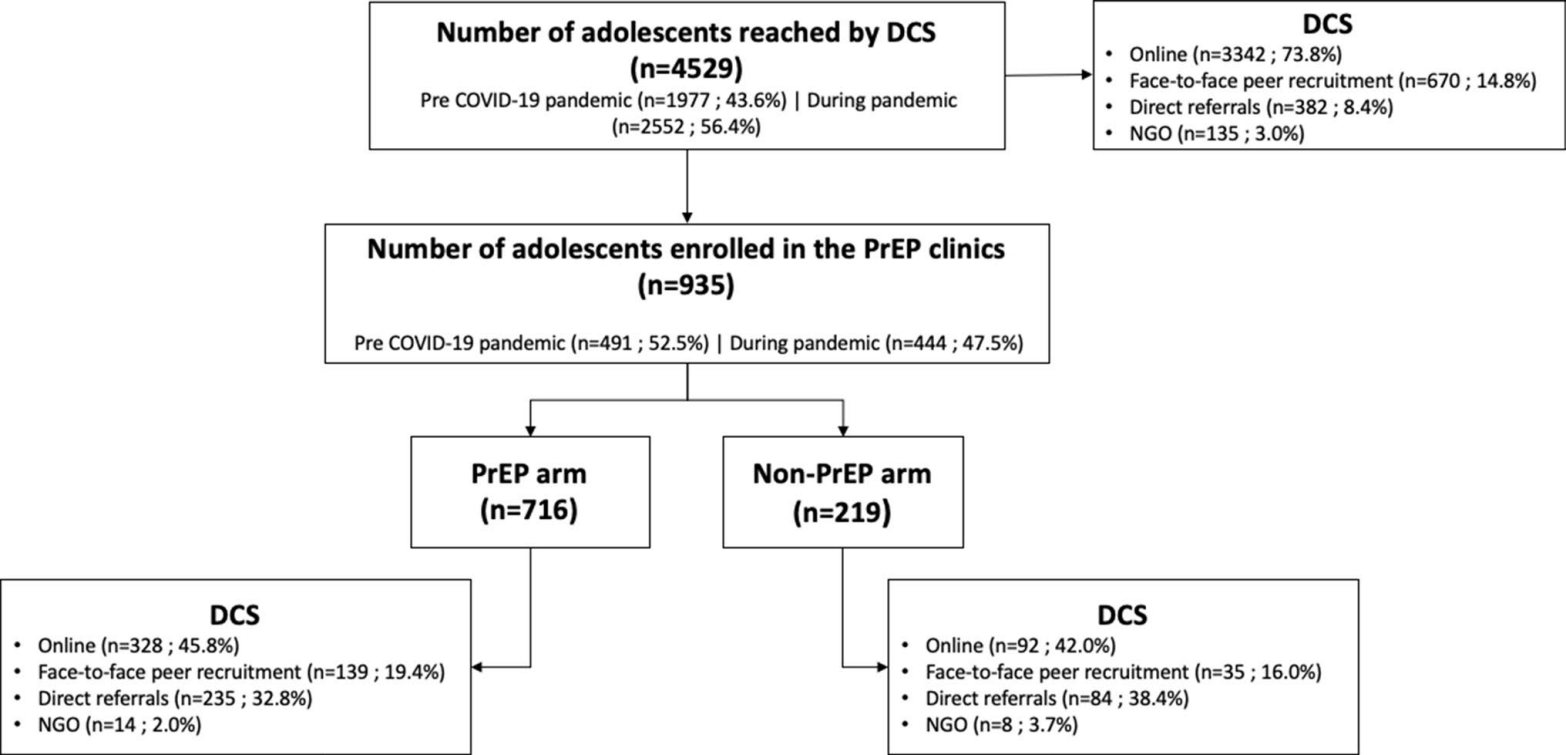
Consort-type diagram of AMSM and ATGW recruitment and enrollment in PrEP and non-PrEP. *DCS—demand creation strategy; PrEP—pre-exposure prophylaxis

Overall, 4,529 AMSM and ATGW were reached by the DCS, most of them via online strategies (73.8%), followed by face-to-face peer recruitment (14.8%), direct referrals (8.4%), and NGO recruitment (3.0%) (Fig. 2). During the COVID-19 pandemic period, there was a substantial increase in online recruitment (from 31.3 to 68.7%) and a decrease in face-to-face recruitment (from 78.1 to 21.9%), direct referrals (from 72.0 to 28.0%), and NGO strategies (from 99.3 to 0.7%) (Table 2).

**Table 2:** AMSM and ATGW reached and enrolled by DCS in PrEP and non-PrEP arm

DCS	Overall population reached							Population enrolled in PrEP							Population enrolled in non-PrEP arm						
	Total (N = 4529)		Pre-COVID-19 (N = 1977; 43.6%)		During COVID-19 (N = 2,552; 56.4%)		*P* value	Total (N = 716)		Pre-COVID-19 (N = 349; 48.7%)		During COVID-19 (N = 367; 51.3%)		*P* value	Total (N = 219)		Pre-COVID-19 (N = 130; 59.4%)		During COVID-19 (N = 89; 40.6%)		*P* value
	n	%	n	%	n	%		n	%	n	%	n	%		n	%	n	%	n	%	
Online	3342	73.8	1045	31.3	2297	68.7	< 0.001	328	45.8	110	33.5	218	66.5	< 0.001	92	42.0	25	27.2	67	72.8	< 0.001
Amanda Selfie	139	4.2	64	46.0	75	54.0		17	5.2	13	76.5	4	23.5		3	3.3	2	66.7	1	33.3	
Peer recruitment on “hook-up” apps	2710	81.1	935	34.5	1775	65.5		221	67.4	79	35.7	142	64.3		49	53.3	18	36.7	31	63.3	
Peer recruitment on Instagram	342	10.2	28	8.2	314	91.8		57	17.4	13	22.8	44	77.2		34	37.0	5	14.7	29	85.3	
Peer recruitment on WhatsApp	151	4.5	18	11.9	133	88.1		33	10.1	5	15.2	28	84.8		6	6.5	0	0.0	6	100.0	
Face-to-face peer recruitment	670	14.8	523	78.1	147	21.9		139	19.4	67	48.2	72	51.8		35	16.0	28	80.0	7	20.0	
School	24	3.6	24	100.0	0	0.0		5	3.6	5	100.0	0	0.0		3	8.6	3	100.0	0	0.0	
Party	112	16.7	109	97.3	3	2.7		13	9.4	12	92.3	1	7.7		3	8.6	3	100.0	0	0.0	
Public venue	376	56.1	355	94.4	21	5.6		59	42.4	48	81.4	11	18.6		21	60.0	20	95.2	1	4.8	
STI clinics	60	9.0	8	13.3	52	86.7		37	26.6	2	5.4	35	94.6		5	14.3	1	20.0	4	80.0	
Others	98	14.6	27	27.6	71	72.4		25	18.0	0	0.0	25	100.0		3	8.6	1	33.3	2	66.7	
Direct referrals	382	8.4	275	72.0	107	28.0		235	32.8	159	67.7	76	32.3		84	38.4	69	82.1	15	17.9	
Communication initiatives in general	56	14.7	50	89.3	6	10.7		22	9.4	17	77.3	5	22.7		11	13.1	11	100.0	0	0.0	
By the health service	26	6.8	14	53.8	12	46.2		22	9.4	13	59.1	9	40.9		1	1.2	0	0.0	1	100.0	
By the participant to a friend or sexual partner	131	34.3	72	55.0	59	45.0		98	41.7	57	58.2	41	41.8		23	27.4	12	52.2	11	47.8	
STI clinic	169	44.2	139	82.2	30	17.8		93	39.6	72	77.4	21	22.6		49	58.3	46	93.9	3	6.1	
NGO	135	3.0	134	99.3	1	0.7		14	2.0	13	92.9	1	7.1		8	3.7	8	100.0	0	0.0	

Of the total number of individuals reached by the DCS, 20.6% (935/4529) were enrolled in the PrEP1519 study; of those, 76.6% (716/935) started using PrEP, and 23.4% (219/935) chose other HIV prevention methods (non-PrEP arm). The DCS that enrolled most adolescents in PrEP and non-PrEP was via online methods (328/716 and 92/219, respectively) and direct referrals (235/716 and 84/219, respectively). However, in terms of effectiveness, enrolling adolescents in the PrEP arm was greater via direct referrals (235/382) and face-to-face recruitment (139/670) than online strategies (328/3342). In the same way, regarding enrolling adolescents in the non-PrEP arm, direct referrals (84/382) and face-to-face recruitment (35/670) were more effective than online strategies were (92/3,342) (Table 2).

Most adolescents enrolled in the PrEP1519 study were enrolled in the São Paulo site (47.7%) and before the COVID-19 pandemic (52.5%). Furthermore, most were AMSM (92.0%); had a lower (34.6%) and middle (38.8%) socioeconomic status; had 12 or more years of schooling (52.1%); self-identified as black skin color (68.0%); were aged between 18 and 19 years (79.9%); were living with parents or relatives (80.1%); and did not participate in organized social movements or LGBTQI + NGO (88.0%). As for their sexual behavior, 21.4% reported having had an STI in the previous 12 months, 32.9% reported drug or alcohol use before or during sex, and 14.3% reported exchanging sex for money or favors. Moreover, most reported condomless anal sex in the past 3 months (79.3%), had their sexual debut at age 15 or under (62.8%); had not used a condom during their first sexual experience (54.2%); and reported prior PrEP knowledge (71.4%). Most had a perception of the moderate risk of HIV infection (46.1%). Concerning experiences of violence and discrimination, 33.0% said they experienced some form of discrimination or violence due to their sexual orientation or gender identity, and 28.5% reported an episode of sexual violence (Table 3).

**Table 3:** Bivariate analysis of the association between sociodemographics, recruitment during the COVID-19 pandemic, sexual behavior, HIV risk perception, discrimination, violence, and demand creation strategies

	Total (N = 935)		Population enrolled in PrEP (N = 716) ^1^									Population enrolled in non-PrEP arm (N = 219) ^1^								
			Online (N = 328)		Referrals (N = 235)		Face-to-face peer recruitment (N = 139)		NGO (N = 14)		*P* value^4^	Online (N = 92)		Referrals (N = 84)		Face-to-face peer recruitment (N = 35)		NGO (N = 8)		*P* value^4^
	n	%	n	%	n	%	n	%	n	%		n	%	n	%	n	%	n	%	
*Sociodemographics and COVID-19 pandemic*																				
Population																				
AMSM	860	92.0	316	48.4	207	31.7	120	18.4	10	1.5	< 0.001	90	43.5	77	37.2	34	16.4	6	2.9	0.0276
ATGW	75	8.0	12	19.0	28	44.4	19	30.2	4	6.3		2	16.7	7	58.3	1	8.3	2	16.7	
Study site																				
Salvador	293	31.3	113	53.1	57	26.8	43	20.2	0	0.0	< 0.001	50	62.5	16	20.0	14	17.5	0	0.0	< 0.001
São Paulo	446	47.7	194	57.4	105	31.1	26	7.7	13	3.8		40	37.0	53	49.1	7	6.5	8	7.4	
Belo Horizonte	196	21.0	21	12.7	73	44.2	70	42.4	1	0.6		2	6.5	15	48.4	14	45.2	0	0.0	
Recruitment during the COVID-19 pandemic																				
No	491	52.5	113	31.3	162	44.9	73	20.2	13	3.6	< 0.001	25	19.2	69	53.1	28	21.5	8	6.2	< 0.001
Yes	444	47.5	215	60.6	73	20.6	66	18.6	1	0.3		67	75.3	15	16.9	7	7.9	0	0.0	
Socioeconomic Status^2^																				
Low	308	34.6	48	21.6	109	49.1	53	23.9	12	5.4	< 0.001	14	16.3	46	53.5	20	23.3	6	7.0	< 0.001
Middle	346	38.8	151	54.5	76	27.4	48	17.3	2	0.7		46	66.7	19	27.5	4	5.8	0	0.0	
High	237	26.6	114	59.1	42	21.8	37	19.2	0	0.0		26	59.1	11	25.0	6	13.6	1	2.3	
Years of schooling																				
1–7	25	2.7	10	47.6	5	23.8	5	23.8	1	4.8	0.0464	2	50.0	1	25.0	1	25.0	0	0.0	0.1517
8–11	423	45.2	136	42.1	111	34.4	65	20.1	11	3.4		45	45.0	33	33.0	15	15.0	7	7.0	
12 or more	487	52.1	182	48.9	119	32.0	69	18.5	2	0.5		45	39.1	50	43.5	19	16.5	1	0.9	
Race/Skin color																				
Non-Black	299	32.0	105	47.9	75	34.2	33	15.1	6	2.7	0.1906	33	41.2	31	38.8	13	16.2	3	3.8	1.00
Black	636	68.0	223	44.9	160	32.2	106	21.3	8	1.6		59	42.4	53	38.1	22	15.8	5	3.6	
Age group																				
15–17 years old	188	20.1	59	40.7	44	30.3	41	28.3	1	0.7	0.0218	20	46.5	15	34.9	7	16.3	1	2.3	0.9085
18–19 years old	747	79.9	269	47.1	191	33.5	98	17.2	13	2.3		72	40.9	69	39.2	28	15.9	7	4.0	
Lives with^3^																				
Parents or relatives	713	80.1	261	47.3	175	31.7	114	20.7	2	0.4	< 0.001	70	43.5	59	36.6	27	16.8	5	3.1	0.4078
Alone, friends, or partners	177	19.9	52	37.4	52	37.4	23	16.5	12	8.6		16	42.1	17	44.7	3	7.9	2	5.3	
Participates in organized social movement or LGBTQI + NGO^3^																				
No	782	88.0	286	47.2	192	31.7	121	20.0	7	1.2	< 0.001	73	41.5	71	40.3	28	15.9	4	2.3	0.0269
Yes	107	12.0	25	29.8	35	41.7	17	20.2	7	8.3		13	56.5	5	21.7	2	8.7	3	13.0	
*Sexual behavior*																				
Reported STI in previous 12 months^3^																				
No	710	78.6	248	46.1	174	32.3	104	19.3	12	2.2	0.2504	67	39.0	67	39.0	31	18.0	7	4.1	0.5449
Yes	193	21.4	58	38.4	61	40.4	30	19.9	2	1.3		20	47.6	17	40.5	4	9.5	1	2.4	
Use of drugs and/or alcohol before or during sex^3^																				
No	606	67.1	204	45.0	157	34.7	82	18.1	10	2.2	0.6644	62	40.5	60	39.2	25	16.3	6	3.9	1.0
Yes	297	32.9	102	43.2	78	33.1	52	22.0	4	1.7		25	41.0	24	39.3	10	16.4	2	3.3	
Sex in exchange for money or favors^3^																				
No	774	85.7	269	46.2	192	33.0	109	18.7	12	2.1	0.1364	76	39.6	75	39.1	34	17.7	7	3.6	0.3587
Yes	129	14.3	37	34.6	43	40.2	25	23.4	2	1.9		11	50.0	9	40.9	1	4.5	1	4.5	
Condomless anal sex in the past 3 months																				
No	194	20.7	62	52.5	37	31.4	19	16.1	0	0.0	0.1763	32	42.1	28	36.8	14	18.4	2	2.6	0.87
Yes	741	79.3	266	44.5	198	33.1	120	20.1	14	2.3		60	42.0	56	39.2	21	14.7	6	4.2	
Age at sexual debut ^3^																				
≤ 15	544	62.8	183	42.3	147	33.9	95	21.9	8	1.8	0.2068	54	48.6	36	32.4	16	14.4	5	4.5	0.0735
> 15	322	37.2	121	49.8	74	30.5	42	17.3	6	2.5		25	31.6	38	48.1	14	17.7	2	2.5	
Used condom at sexual debut^3^																				
No	479	54.2	167	44.2	128	33.9	74	19.6	9	2.4	0.8095	49	48.5	35	34.7	15	14.9	2	2.0	0.3123
Yes	405	45.8	143	46.4	97	31.5	63	20.5	5	1.6		36	37.1	41	42.3	15	15.5	5	5.2	
Prior knowledge of PrEP^3^																				
No	249	28.6	85	45.9	60	32.4	37	20.0	3	1.6	0.9002	19	29.7	31	48.4	13	20.3	1	1.6	0.1089
Yes	622	71.4	207	42.8	165	34.1	101	20.9	11	2.3		62	44.9	48	34.8	22	15.9	6	4.3	
Acceptability of PrEP																				
No	123	13.2	10	66.7	5	33.3	0	0.0	0	0.0	0.1607	49	45.4	37	34.3	18	16.7	4	3.7	0.6617
Yes	812	86.8	318	45.4	230	32.8	139	19.8	14	2.0		43	38.7	47	42.3	17	15.3	4	3.6	
*HIV risk perception*																				
Perceived risk for HIV infection^3^																				
Low	295	33.4	92	41.3	79	35.4	51	22.9	1	0.4	0.1044	25	34.7	34	47.2	11	15.3	2	2.8	0.0198
Moderate	407	46.1	150	48.2	99	31.8	52	16.7	10	3.2		40	41.7	34	35.4	18	18.8	4	4.2	
High	180	20.4	68	45.0	46	30.5	34	22.5	3	2.0		21	72.4	6	20.7	1	3.4	1	3.4	
*Discrimination and violence*																				
Ever experienced violence or discrimination related to sexual orientation or gender identity^3^																				
No	605	67.0	211	47.6	153	34.5	74	16.7	5	1.1	0.0054	69	42.6	66	40.7	22	13.6	5	3.1	0.1641
Yes	298	33.0	95	38.6	82	33.3	60	24.4	9	3.7		18	34.6	18	34.6	13	25.0	3	5.8	
Ever experienced sexual violence^3^																				
No	629	71.5	217	45.6	166	34.9	85	17.9	8	1.7	0.0967	61	39.9	61	39.9	26	17.0	5	3.3	0.1597
Yes	251	28.5	92	44.2	59	28.4	51	24.5	6	2.9		24	55.8	14	32.6	3	7.0	2	4.7	

^1^Outcome was DCS in four categories (online, face-to-face peer recruitment, direct referrals, and NGO)

^2^Calculated using latent class analysis

^3^Contains missing values

^4^*P* values obtained through the Fisher test

In the bivariate analysis of predictors of DCS across the four categories, we observed that online strategies resulted in the recruitment of the following profile of enrollment in the PrEP arm: AMSM (48.4%), from the São Paulo site (57.4%), recruited during the COVID-19 pandemic period (60.6%), with a high socioeconomic status (59.1%), with 12 years or more of education (48.9%), and aged 18 or 19 years (47.1%). On the other hand, direct referrals played an important role in recruiting for PrEP enrollment, before the COVID-19 pandemic period (44.9%) and reached a higher proportion of TGW (44.4%) and participants with lower socioeconomic status (49.1%). In the non-PrEP arm, the online strategies played a part in enrolling a higher proportion of AMSM (43.5%), from the Salvador site (62.5%), during the COVID-19 pandemic period (75.3%), reaching those with a higher socioeconomic status (59.1%), and who had a high-HIV risk perception (72.4%). In addition, direct referrals resulted in a higher proportion of TGW (58.3%) being recruited for the non-PrEP arm, before the COVID-19 pandemic period (53.1%), reaching participants with lower socioeconomic status (53.5%) and low perception of an HIV risk (47.2%) (Table 3).

Table 4 shows the bivariate and multivariate analyses of factors associated with DCS comparing online and face-to-face strategies for enrollment. In the multivariate analysis, the odds of being enrolled in PrEP through an online strategy was greater among AMSM (aOR = 3.65; 95%CI: 1.74–8.28), those with middle (aOR = 3.24; 95%CI: 1.61–6.63) and higher socioeconomic levels (aOR = 3.36; 95%CI: 1.34–8.95), living with parents or relatives (aOR = 1.63; 95%CI: 1.02–2.62), and during the COVID-19 pandemic (aOR = 1.67; 95%CI: 1.06–2.61). Furthermore, the odds of being enrolled in the non-PrEP arm were greater among those with middle and higher socioeconomic levels (aOR = 3.22; 95%CI: 1.10–9.55; aOR = 4.23; 95%CI: 1.25–14.63, respectively), those who had a high-HIV risk perception (aOR = 4.32; 95%CI: 1.32–15.35), and during the COVID-19 pandemic (aOR = 6.32; 95%CI: 2.36–17.76).

**Table 4:** Bivariate and multivariate analysis of the association between sociodemographics, recruitment during the COVID-19 pandemic, sexual behavior, HIV risk perception, discrimination, violence, and demand creation strategies comparing online and face-to-face strategy for enrollment, Brazil (2019–21)

Variables	PrEP arm ^1^ (N = 716)					Non-PrEP arm ^2^ (N = 219)				
	Bivariate analysis^3^			Multivariate analysis^1, 3, 4^		Bivariate analysis^3^			Multivariate analysis^2, 3, 4^	
	OR	95% CI	*P* value	OR	95% CI	OR	95% CI	*P* value	OR	95% CI
*Sociodemographics and COVID-19 pandemic*										
Population										
ATGW	1			1		1				
AMSM	3.99	[2.16–7.96]	< 0.0001	3.65	[1.74–8.28]	3.85	[0.98–25.43]	0.0870		
Study site										
Salvador	1					1				
São Paulo	1.19	[0.84–1.68]	0.318			0.35	[0.19: 0.64]	0.0006		
Belo Horizonte	0.13	[0.07: 0.22]	< 0.0001			0.04	[0.01–0.15]	< 0.0001		
Socioeconomic status^5^										
Low	1			1		1			1	
Middle	4.34	[2.94–6.51]	< 0.0001	3.24	[1.61–6.63]	10.29	[4.93–22.68]	< 0.0001	3.22	[1.10–9.55]
High	5.23	[3.43–8.09]	< 0.0001	3.36	[1.34–8.95]	7.43	[3.30–17.50]	< 0.0001	4.23	[1.25–14.63]
Years of schooling										
1–7	1					1				
8–11	0.80	[0.33–1.97]	0.621			0.82	[0.10: 7.04]	0.844		
12 or more	1.05	[0.43–2.59]	0.907			0.64	[0.07: 5.51]	0.664		
Race/Skin color										
Non-Black	1					1				
Black	0.88	[0.64–1.22]	0.447			1.05	[0.60–1.84]	0.863		
Age group										
15–17 years old	1					1				
18–19 years old	1.30	[0.90: 1.89]	0.1665			0.80	[0.41: 1.57]	0.505		
Lives with ^6^										
Alone, friends, or partners	1			1		1				
Parents or relatives	1.5	[1.03: 2.21]	0.03732	1.63	[1.02–2.62]	1.06	[0.52–2.19]	0.878		
Participates in organized social movement or LGBTQI + NGO^6^										
No	1					1				
Yes	0.47	[0.28–0.77]	0.00308			1.83	[0.77–4.52]	0.1753		
Recruitment during the COVID-19 pandemic										
No	1			1		1			1	
Yes	3.37	[2.48: 4.60]	< 0.0001	1.67	[1.06–2.61]	12.79	[6.80–25.02]	< 0.0001	6.32	[2.36–17.76]
*Sexual behavior*										
Reported STI in the past 12 months^6^										
No	1					1				
Yes	0.73	[0.50–1.05]	0.0937			1.42	[0.72–2.81]	0.30669		
Condomless anal sex in the past 3 months										
No	1					1				
Yes	0.72	[0.49–1.07]	0.109			0.99	[0.57–1.75]	0.983		
Use of drugs and/or alcohol before or during sex^6^										
No	1					1				
Yes	0.93	[0.68–1.28]	0.6495			1.02	[0.55–1.86]	0.9506		
Sex in exchange for money or favors^6^										
No	1					1				
Yes	0.62	[0.40–0.94]	0.0269			1.53	[0.62–3.74]	0.3487		
Used condom at sexual debut^6^										
No	1					1				
Yes	1.10	[0.81–1.48]	0.5561			0.63	[0.35–1.10]	0.106		
Age at sexual debut^6^										
≤ 15	1					1				
> 15	1.35	[ 0.99–1.86]	0.0592			0.49	[0.26–0.89]	0.0199		
Prior knowledge of PrEP^6^										
No	1					1				
Yes	0.88	[0.63–1.24]	0.459			1.93	[1.04–3.70]	0.04126		
Acceptability of PrEP										
No	1					1				
Yes	0.42	[0.13–1.18]	0.112			0.76	[0.44–1.30]	0.321		
*HIV risk perception*										
Perceived risk for HIV infection^6^										
Low	1					1			1	
Moderate	1.33	[0.94–1.88]	0.1106			1.34	[0.72–2.55]	0.3610	1.24	[0.51–3.06]
High	1.17	[0.77–1.77]	0.4689			4.93	[1.97–13.38]	0.0010	4.32	[1.32–15.35]
*Discrimination and violence*										
Ever experienced violence or discrimination related to sexual orientation or gender identity^6^										
No	1					1				
Yes	0.69	[0.50–0.95]	0.0228			0.71	[0.37–1.35]	0.3093		
Ever experienced sexual violence^6^										
No	1					1				
Yes	0.95	[0.68–1.31]	0.7428			1.91	[0.96–3.81]	0.0645		

^1^Face-to-face, n = 388; Online, n = 328, Multivariate model, n = 691

^2^Face-to-face, n = 127; Online, n = 92, Multivariate model, n = 197

^3^Outcome was DCS recategorized into “online” (only online peer recruitment) and “face-to-face” (face-to-face peer recruitment, direct referrals, and NGO)

^4^Models adjusted by study site and interaction between study site and socioeconomic class

^5^Calculated using latent class analysis

^6^Contains missing values

## Discussion

Our results demonstrate that the different DCS reached different profiles of AMSM and ATGW. Although online DCS reached a higher number of adolescents, it were less efficient in recruiting adolescents who were actually enrolled in PrEP than face-to-face strategies, which were also more capable of recruiting those at higher socioeconomic vulnerability. Moreover, we highlight the increase in the adolescents reached through online strategies and the decrease in face-to-face strategies during the COVID-19 pandemic, which resulted in a higher proportion of enrollments for PrEP and non-PrEP via online strategies during this period.

The COVID-19 pandemic may have contributed to an increase in the vulnerability of AMSM and ATGW to HIV infection (Grangeiro et al., 2021) because it affected their access to HIV prevention and testing services in various countries (Rao et al., 2021; Sanchez et al., 2020). Nevertheless, in our study, the proportion of enrolled participants was similar (52.5% pre- and 47.5% during the COVID-19 pandemic) and the PrEP1519 contingency plan, which included the intensification of online strategies during the pandemic, may have contributed to that result. Based on this contingency plan, PrEP1519 clinics continued their work during the pandemic and were able to adapt quickly to the new situation using a social media and telemonitoring infrastructure (Dourado et al., 2020; Magno et al., 2021).

The adolescents enrolled in the PrEP1519 study had a high social vulnerability, low socioeconomic status, and often engaged in high-risk sexual behavior. Unlike our study, in the Brazilian National Health System (in Portuguese: *Sistema Único de Saúde–SUS*) PrEP Program, where PrEP is only available for individuals aged 18 or above (as of 2022, PrEP became available for people over 15 years old), most PrEP users are white, with a high level of schooling, and aged between 30 and 39 years (Brasil, 2020b). The intersection of race, sexuality, and age is important because discrimination against black male adolescents is a well-documented reality in Brazil, especially in schools, and generally manifests indirectly in social relations, with individuals being differentiated according to their ethnic background (Guimarães & Pinto, 2016; Magno et al., 2022). The high enrollment of participants with black skin color in the PrEP1519 study differs from studies with adolescents carried out in the USA (Bradley et al., 2020).

ATGW are harder to engage both in PrEP and other HIV combination prevention methods when compared to AMSM. This suggests that despite efforts made using different DCS, the high social vulnerability of TGW and the social context of stigma and discrimination may have influenced their access to the study (Magno et al., 2019a, b). Other Brazilian studies have shown that TGW face discrimination in various spheres of their lives from adolescence onward, and tend to have limited access to education/training, formal employment, and social services in general, especially health services (Magno et al., 2018a, b; Soares et al., 2019).

The higher number of adolescents reached by the online DCS can be explained as the result of using a strategy that is compatible with the social dynamics of young people. It is also noteworthy that almost 81% of all online recruitment was conducted through dating or “hook-up” apps, which demonstrates that adolescents are more willing to talk about prevention in these spaces because they are at a greater risk of HIV infection (Chan et al., 2018). Online platforms and app-based prevention strategies were found as important tools to disseminate knowledge and promote healthier attitudes and practices among young people around the world (Maloney et al., 2020; Sullivan & Hightow-Weidman, 2021; Whiteley et al., 2018). In our study, the online strategy mediated by peer educators attracted the attention of and enrolled adolescents at high risk of HIV in PrEP and other combination prevention strategies.

The main limitation of the online DCS is that it tended to attract those AMSM with higher levels of schooling and income. This result can be explained by the unequal access to digital technologies in Brazil, such as computers, tablets, smartphones, and internet connection (Nishijima et al., 2017). A Chicago-based study with MSM analyzing online youth recruitment for HIV prevention found that Black and Latino youth used the internet less than their white peers, and that blacks were less likely to be recruited online than whites (Du Bois et al., 2012). This unequal access to digital technologies, known as the digital divide, affects several marginalized population groups (Chesser et al., 2016).

In this sense, studies have shown that face-to-face strategies and referrals from health services play a crucial role in reaching out to adolescents with greater social vulnerability, as we saw in our findings. During the implementation of a test-and-treat and HIV prevention program in Thailand in 2015 and 2016, peer-driven recruitment was effective for recruiting MSM and TGW living with HIV for treatment, and in recruiting MSM and TGW at high risk for HIV infection (Ongwandee et al., 2018).

Only one-fifth of the participants approached joined the study. This indicates that reaching out to and creating demand for HIV prevention among AMSM and ATGW requires a broad view that takes into account the diversity of individuals, their access to HIV prevention methods, health services, and the structural and political scenario underlying the HIV response. In contexts of growing conservatism in Brazil in the recent past and present day, comprehensive sex education has been undermined at schools.

An overwhelming silence about HIV and STI can be observed in the mainstream media. In recent years, issues pertaining to sex are not discussed at home or at school, primarily because of the rise of religious and political conservatism (Paiva et al., 2020; Reis Brandão & Cabral, 2019). In other LMIC, sexual health education programs for adolescents are still a challenge (DeMaria et al., 2009; Mashora et al., 2019; Melesse et al., 2020), which means this educational void tends to be filled by the internet (Burki, 2016). Furthermore, when an adolescent’s sexual orientation or gender identity does not conform to prevailing cisgender and heteronormative standards, they tend to be more vulnerable socially and programmatically, which is further aggravated by stigma and discrimination (Hatzenbuehler & Pachankis, 2016; Peng et al., 2019).

Therefore, an important factor for encouraging the engagement of AMSM and ATGW in HIV prevention services is to provide friendly services, free of sexuality- and gender-related stigma and discrimination. Consequently, considering the context of the three study sites, recruiting and enrolling AMSM and ATGW in a PrEP demonstration study in this scenario required the development of a variety of DCS. As such, face-to-face DCS of the PrEP1519 study can also be seen as interventions that created physical spaces, networks, and social gathering; used networks as well as personal and social competences/skills of participants and peer educators; used preexisting networks and activities in the community, and promoted community-based HIV testing in youth hotspots.

The limits of this study also include the convenience sampling method and the inclusion of the COVID-19 pandemic period, which disrupted face-to-face DCS, which could explain why more people were reached via online strategies. Furthermore, most of the study population were 18–19 years of age, which may limit generalization to younger adolescents. Nonetheless, the study was able to develop and analyze DCS that enrolled a diverse group of AMSM and ATGW, indicating that they are interested in PrEP and other HIV combination prevention once they are made aware of the same.

### Conclusion

Recruiting AMSM and ATGW with different socioeconomic and behavioral profiles for HIV prevention services requires a combination of DCS. Our study points out the need for DCS that do not solely depend on spontaneous demand from health services, but which actively reach out to adolescents among populations at the greatest risk for HIV infection, while respecting their specific needs and choices and adopting local strategies for demand creation, recruitment, monitoring, and trained health care providers and peer educators. Moreover, the findings have been shared with the Brazilian Ministry of Health, with the aim to help in the establishment of a comprehensive network of public services which are able to tackle the particularities and needs of this highly vulnerable population, which includes, but is not limited, to stigma-free access to all HIV/STI combination prevention strategies.

## Data Availability

Data and materials cannot be shared publicly because of their sensitive content. The informed consent process prior to participation ensured confidentiality and anonymity and that only investigators of the PrEP1519 project would be allowed to access the data collected. With these conditions assured, ethical approval was obtained from the Research Ethics Review Committees of the Universidade de São Paulo (protocol number 70798017.3.0000.0065), Universidade Federal da Bahia (protocol number 01691718.1.0000.5030), and Universidade Federal de Minas Gerais (protocol number 17750313.0.0000.5149). In the best interest of protecting participants’ confidentiality and anonymity, researchers may contact the Research Ethics Committee of Universidade de São Paulo, (Comissão de Ética para Análise de Projetos de Pesquisa, email: cappesq.adm@hc.fm.usp.br), to make requests related to access to the data used for the analyses in this manuscript.

## Abbreviations

AF: Assent form
AMSM: Adolescent men who have sex with men
aOR: Adjusted odds ratio
ATGW: Adolescent transgender women
DCS: Demand creation strategies
LCA: Latent class analysis
LGBTQI +: Lesbian, gay, bisexual, transgender, queer, intersexual, and others
NGO: Non-governmental organization
PrEP: HIV pre-exposure prophylaxis
STI: Sexually transmitted infections
WIC: Written informed consent
USP: University of São Paulo
UFBA: Federal University of Bahia
UFMG: Federal University of Minas Gerais
